# Incidence and predictors of tuberculosis among children receiving antiretroviral therapy in the Wolaita Zone: A retrospective cohort study

**DOI:** 10.1371/journal.pone.0291502

**Published:** 2023-09-21

**Authors:** Daneil Tekese, Desalegn Dawit, Behailu Hawulte, Hussein Mohammed, Fekede Asefa, Lemessa Oljira

**Affiliations:** 1 Wolaita Zone Health Department, Wolaita Sodo, Wolaita Zone, Ethiopia; 2 Departemt of Public Health, College of Medicine and Health Sciences, Hawassa University, Hawassa, Ethiopia; 3 School of Public Health, College of Health and Medical Sciences, Haramaya University, Harar, Ethiopia; 4 Department of Pediatrics, The University of Tennessee Health Science Center (UTHSC)—Oak Ridge National Laboratory (ORNL) Center for Biomedical Informatics, College of Medicine, Memphis, TN, United States of America; Madda Walabu University, ETHIOPIA

## Abstract

**Background:**

Tuberculosis is the leading cause of morbidity and mortality among children living with the human immunodeficiency virus (HIV), mainly in sub-Saharan Africa, including Ethiopia. Tuberculosis remains a significant health concern for HIV-positive children in Ethiopia. There is a paucity of data on the incidence and predictors of tuberculosis among children living with HIV on antiretroviral therapy in the Wolaita zone. Hence, this study aimed to assess the incidence and predictors of tuberculosis among children living with HIV on antiretroviral therapy in the Wolaita zone between January 2010 to December 2020.

**Methods:**

A retrospective cohort study was conducted among 389 children receiving antiretroviral therapy in Wolaita zone health facilities between January 2010 to December 2020. The checklist was adapted from the standardized antiretroviral treatment (ART) follow-up form currently used by the institutions’ ART clinics. The Kaplan-Meier survival function and Log-rank were used to estimate the survival for each categorical variable to compare the survival between different exposure groups. Both bivariable and multivariable parametric survival Gompertz models were fitted to identify predictors of tuberculosis among HIV-positive children. The association was summarized using an adjusted hazard ratio (AHR), and statistical significance was declared at 95% CI and p-value < 0.05. The goodness of the model fit was assessed using a Cox-Snell residual plot.

**Results:**

The incidence rate of tuberculosis among children living with HIV was 3.5 (95% CI 2.7–4.5) per 100 child years. World Health Organization clinical stage III or IV (AHR = 2.31, 95% CI [1.26, 4.22]), hemoglobin level <10 g/dL (AHR = 2.87, 95% CI [1.51, 5.45]), fair or poor ART adherence (AHR = 4.4, 95% CI[2.18, 9.05]), underweight (AHR = 2.55, 95% CI [1.45, 4.51]), age >10 years (AHR = 3.62; 95% CI [1.29, 10.0]), and cotrimoxazole preventive therapy (AHR = 0.23; 95% CI [0.08, 0.60]) were among the independent predictors of TB occurrence.

**Conclusion:**

The incidence of tuberculosis among children on ART was high. HIV-positive children presenting with advanced disease staging (III and IV), anemia, “fair” and “poor” ART adherence, underweight, age above ten years, and not receiving cotrimoxazole preventive therapy were at higher risk of TB. Therefore, counseling on ART adherence, early diagnosis, and prompt treatment of anemia and malnutrition are recommended to avert tuberculosis.

## Introduction

Tuberculosis (TB) is the leading cause of morbidity and mortality globally [1]. Tuberculosis incidence varies globally, with 30 high-burden countries accounting for 87% of cases and eight countries having over two-thirds of global cases [2]. In 2021, there were 1.2 million new children’s TB cases, accounting for 12% of the global TB burden [2].

The global tuberculosis mortality rate increased significantly from 1.4 million in 2019 to 1.6 million in 2021, including 187,000 people with HIV [1,2]. HIV infection increases susceptibility to TB infection and is a critical factor in triggering active TB infection [3,4]. Immunosuppression imposed by HIV is responsible for opportunistic infections (OIs), which are the leading cause of morbidity and mortality in children with HIV around the world [5].

TB is the most common OI among children living with HIV; one in three HIV-positive children develops tuberculosis [6]. In Ethiopia, the incidence rate of tuberculosis among children receiving ART is high across different settings. The incidence rate of tuberculosis among HIV-positive children receiving ART ranged from 2.63 per 100 child-years of observation at Debre-Markos Referral Hospital, northwest Ethiopia [7] to 7.9 per 100 child-years of observation in Southwest Ethiopia [8].

Studies reported that several factors, such as advanced WHO clinical staging (III or IV) [7,9], poor ART drug adherence [7], not receiving isoniazid preventive therapy (IPT) [9,10], absence of cotrimoxazole preventive therapy (CPT) [9], anemia (Hgb < 10 g/dL) [9,10], low CD4 count [9,11], and malnutrition [9,10] were significantly increasing the risk of incidence of TB among children receiving ART. However, further studies are needed to investigate potential contextual variations in factors that increase the risk of TB among children living with HIV.

The global community launched the End TB strategy in 2015. Sustainable Development Goals were planned in 2015 to reduce TB-related deaths by 75% in 2025 and 90% by 2030 [12]. Averting tuberculosis is crucial for improving HIV survival in resource-limited countries, and Ethiopia is nearing milestones in the end-TB strategy for reduced tuberculosis incidence and deaths [2].

Isoniazid preventive therapy (IPT) is considered the most vital intervention to avert TB incidence among people living with HIV, including children, and has been recommended as a component of a comprehensive HIV/AIDS care strategy [13]. However, a study conducted by Wasie and Tigabu in 2018 at two referral hospitals in northwest Ethiopia reported that only 37% of children living with HIV received IPT [14]. Moreover, previous studies have produced contradictory findings regarding the impact of IPT on TB incidence among children living with HIV. Some studies have indicated that IPT significantly reduces TB incidence among children with HIV in Ethiopia [9,10,15], while others have found no significant reduction in TB incidence [6,7,16]. This inconclusiveness underscores the need for further investigation. Furthermore, there is a paucity of data on the incidence and predictors of tuberculosis among children living with HIV on antiretroviral therapy in the Wolaita Zone. This study aims to assess the incidence and predictors of tuberculosis among children receiving antiretroviral therapy in the Wolaita Zone.

## Methods and materials

### Study design and setting

A retrospective cohort study was conducted among HIV-positive children enrolled at Wolaita Zone health facilities from January 2010 to December 2020. The Wolaita zone is one of the administrative zones in the southern nations and nationalities region, and it has 16 districts and eight town administrations. In this zone are seven hospitals, five governmental hospitals, one private hospital, one non-governmental hospital, 68 governmental health centers, and two non-governmental health centers that render preventive, curative, and rehabilitative services. Among the aforementioned facilities, seven health centers and all hospitals provide ART services in the Wolaita zone. A total of 389 children were enrolled in HIV care in Wolaita zone health facilities from January 2010 to December 2020.

### Population and sample size determination

All HIV-positive children ages less than 15 who were enrolled in the pediatric HIV care clinic in the Wolaita Zone health facilities and who had at least one month of ART follow-up were the source population. HIV-infected children aged less than 15 who were enrolled in the pediatric HIV care clinic at Wolaita Zone between January 2010 and December 2020 were the study population. Children with HIV who already had TB before starting ART, had incomplete baseline information (CD4 count, hemoglobin level, WHO clinical stage, weight, and height), an unknown date of treatment initiation, and a date of TB occurrence were excluded from the study.

The minimum sample size was determined using an exponential model considering the following assumptions; margin of error of 5%, power of 90%, and underweight [AHR = 1.7] [9]. The calculated sample size after adding a 10% contingency for missing and incomplete data, yields 206. All 389 HIV-infected children aged less than 15 who were enrolled in the Pediatric HIV Care Clinic at Wolaita Zone between January 2010 to December 2020, were included in the study.

power exponential, hratio (1.7) effect(hratio) power (0.9) nratio (2)

### Variables of the study

The outcome variable for this study was the occurrence of tuberculosis during follow-up. The independent variables included socio-demographic characteristics (child age, caregiver age, residence, family size, caregiver relationship to the child, and mother’s HIV status), baseline clinical, nutritional, and laboratory characteristics including (WHO clinical stage, CD4 count/percent, hemoglobin level, and nutritional status), and treatment-related characteristics (ART regimen, adherence, IPT and CPT).

### Operational definitions of variables

**Events**: the occurrence of TB among HIV-infected children during the follow-up period at any time after enrollment to the pediatrics HIV care clinic. TB cases were diagnosed based on the national TB diagnosis guideline, using sputum or gastric aspirate microscopy, chest X-ray examination, and/or histopathology [17].

**Censored**: children who were lost to follow-up, dropped out, transferred out, died due to any causes, or did not develop the events until the last visit.

TB‑free probability time was considered between ART starting and the TB diagnosis date. A loss to follow-up- children missing their appointment for follow-up or drug picks for more than three months.

Transferred out those children who were transferred to other healthcare facilities.

**Adherence to ART**: is classified into good, fair, and poor, according to the percentage of drug dosage calculated from the total monthly dose of ART drugs, which is described as good (≥ 95% or < 2 doses missed per month or < 3 doses missed per 2 months), fair (85–94% or 3–5 doses missed per 30 doses or 3–9 doses of 60 doses), and poor (less than 85% or > 6 doses of 30 doses or > 9 doses of 60 doses) [18].

**Underweight** (children with weight for age Z-score < − 2 standard deviation (SD), stunted (height for age Z-score < − 2 SD), and wasted (weight for height Z-score < − 2 SD) [19]

CD4 cell count below threshold CD4 cell count < 1500/ mm3 (< 25%) for < 12 months, CD4 cell count < 750/mm3 (< 20%) for age 12–35 months, CD4 cell count < 350/ mm3 (< 15%) for age 36–59 months, and CD4 cell count < 200/mm3 (< 15%) for age≥ 60 months [20].

### Data collection methods

The data extraction tool was developed from the WHO standardized ART entry and follow-up form, which the ART clinics currently use. Data were extracted from the patient’s charts, registers, and logbooks. The data were collected by five BSc nurses who took ART training by using ODK and securing the COVID privation strategy (data collectors keep personal distance and use face masks and sanitizers).

### Data quality control

To ensure data quality, one-day training was offered for the data collectors and the supervisor on how to extract the data based on the study objectives. To check the consistency of the data extraction checklist, the pre-test was conducted on 5% of the sample (19 medical records) of children who had recently enrolled in the ART program. The supervisor and principal investigator checked the data for consistency and completeness daily. The codebook contained data cleaning for any missing values and data errors.

### Data processing and analysis

Data were collected using ODK collect version 1.25.2 and then exported to STATA version 16 for analysis. Descriptive statistics, including proportions, medians, tables, and charts, were used to describe the sociodemographic, clinical, and nutritional characteristics of the study participants. The incidence rate of tuberculosis was calculated. The Kaplan-Meier survival function and Log-rank were used to estimate the survival for each categorical variable to compare the survival between different exposure groups. The proportionality assumption was tested by a global test based on Schoenfeld residuals. A parametric survival analysis using Gompertz regression was used to identify predictors of tuberculosis. Variables with a p-value of < 0.25 in the bivariable analysis were fitted into the multivariable Gompertz regression model. An adjusted hazard ratio with its corresponding 95% confidence interval (CI) was reported, and a P-value less than 0.05 was used to declare the presence of a significant association. The goodness of the model fit was assessed using a Cox-Snell residual plot.

### Ethical considerations

Ethical clearance was obtained from the Institutional Health Research Ethics Review Committee of Haramaya University, College of Health and Medical Sciences. Permission letter was received from the Wolaita zone health department, public hospital administrations, and HIV care clinics’ focal persons to use the secondary data for this study. As the study was conducted through a review of patient records, no consent was obtained from the patients. Information about specific personal identifiers like patient names was not collected, and personal information was kept confidential throughout the study.

## Results

### Demographic characteristics

A total of 389 medical records of HIV-positive children on ART were reviewed. Of these, 371 (95.4%) were included in this study, and the remaining 18 (4.6%) were excluded from the analysis due to incompleteness. The mean (SD) age of the study participants was 7.1(±2.65) years, and 259 (69.8%) were found in the age group of 5–10 years. More than half, 216 (58.2%), were male, and 293 (78.9%) were from urban areas. Three hundred thirty-one (84.37%) lived together with their parents, and 159 (41.86%) had more than six family members (Table 1).

**Table 1:** Baseline demographic characteristics of children receiving ART in Wolaita Zone, southern Ethiopia, January 2010 to December 2022.

Variables	Category	Frequency	Per cent
	0–5 year	63	16.9
	5–10 year	259	69.8
	10–15 year	49	13.2
Sex	Male	216	58.2
	Female	155	41.8
Residence	Urban	293	79
	Rural	78	21
Care givers	Parents	313	84.4
	Orphanage centers	11	2.96
	Sibling	32	8.63
	Grand parents	15	4.04
Family size	≤2	36	16.98
	3–5	176	69.81
	≥5	159	13.21

### Baseline clinical, laboratory, and nutritional characteristics

At baseline 279 (75.2%) children were in WHO clinical stages I or II. The median CD4+ count was 226 (IQR = 132–446) cells/l, and 292 (78.8%) had CD4+ counts > 350 cells/l. The median hemoglobin level was 12 (IQR = 11–12.3) mg/dl, and 48 (13%) had a 10 mg/dl hemoglobin level. Seventy-four (20%) were underweight, and 57 (15.4) were stunted at baseline. Three hundred fifty-nine (96.8%) had CPT, and 152 (40.97%) received IPT. Three hundred thirty-nine (91.4) children had a good adherence level to ART (Table 2).

**Table 2:** Baseline clinical, laboratory, and medication-related characteristics of children on ART in Wolaita Zone, southern Ethiopia, January 2010 to December 2022.

Variables	Category	Frequency	Percent
Baseline WHO stage	Stage 1 and 2	279	75.2
	Stage 3 and 4	92	24.8
Baseline Hgb level	< 10 mg/dl	48	13
	≥ 10 mg/dl	323	87
Baseline CD4 count	below threshold	79	21.2
	above threshold	292	78.8
IPT	Yes	318	85.7
	No	53	24.3
CPT	Yes	359	96.8
	No	12	3.2
ART Adherence	Good	339	91.4
	Ever had fair and Poor	32	8.6
HFA at baseline	Not stunted	314	84.6
	Stunted	57	15.4
WFH at baseline	Not wasted	328	88.4
	Wasted	43	11.6
WFA at baseline	Normal	297	80
	Underweight	74	20

WHO: World Health Organization; IPT: Isoniazid preventive therapy; CPT: Cotrimoxazole preventive therapy; HFA: Hight for Age; WFH: Weight for age; WFA: Weight for age.

### Follow-up time and incidence rate of tuberculosis

**Fig 1:**
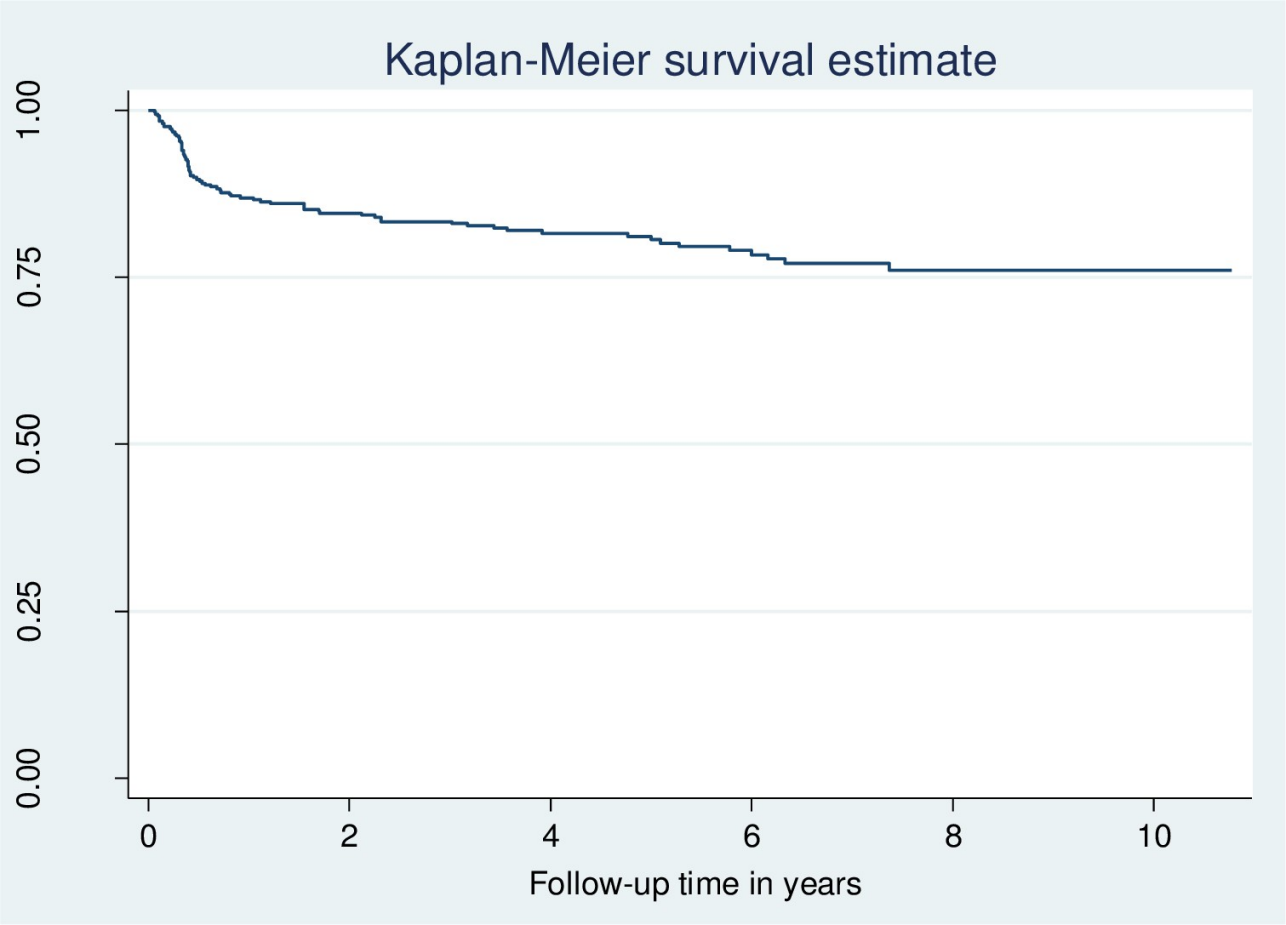
Kaplan-Meier estimate of TB-free probability in children on ART in Wolaita Zone, southern Ethiopia, January 2010 to December 2022.

A total of 371 children followed from 1 to 131 months. The mean follow-up time was 53 months (ranging from 25.8 to 82.6 months). The total time at risk was 1677.5 child years. New TB cases were observed in 59 (15.9%) children; the overall TB incidence rate was 3.5 (95% CI 2.7–4.5) per 100 child years (Fig 1).

**Fig 2:**
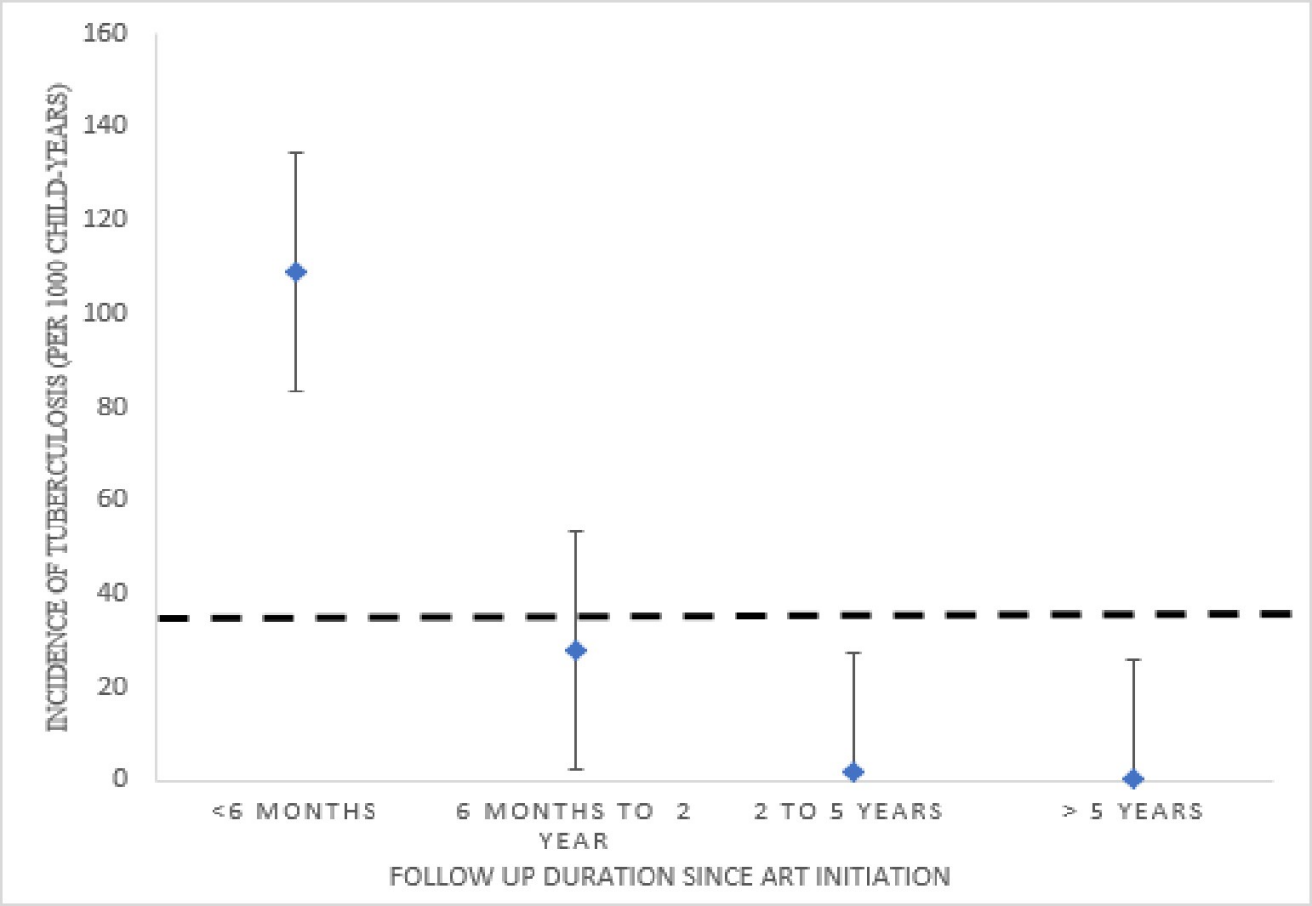
Incidence rates of tuberculosis and their corresponding 95% confidence interval after antiretroviral therapy (ART) initiation, stratified by follow-up duration. Circles: Estimated incidence rates per stratum of follow-up duration since ART initiation. Dotted line: Overall incidence rate.

The incidence rate of TB was 110 (95% CI, 72–166) per 1000 child years in the five months of ART initiation. It sharply decreased to 0.62 (95% CI, 0.32–1.1) per 1000 child years after five years of ART initiation (Fig 2).

**Fig 3:**
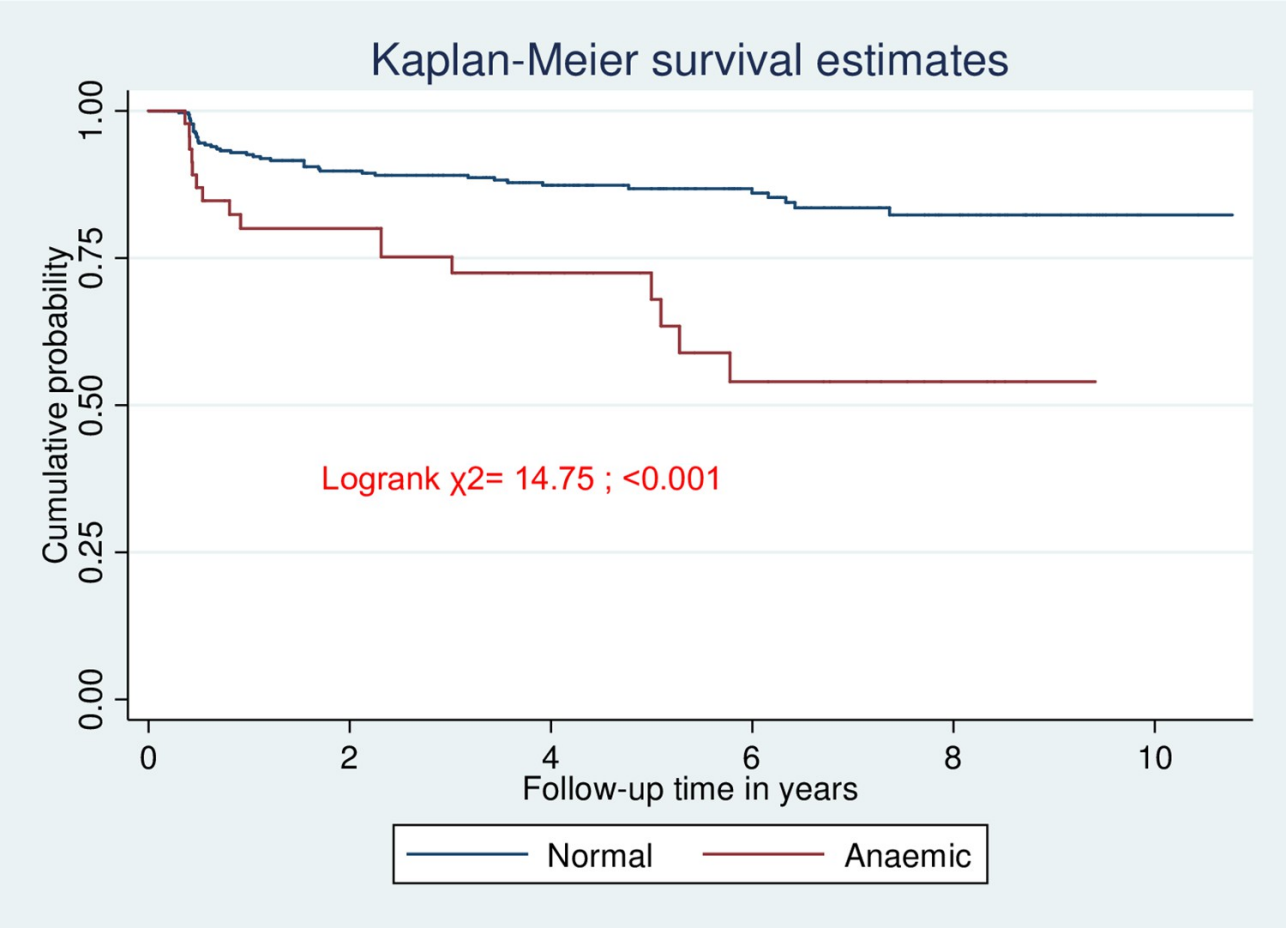
Kaplan-Meier survival estimates for hemoglobin level among children on ART in Wolaita Zone, southern Ethiopia, January 2010 to December 2022.

The incidence rate of TB was 9.0 (95% CI, 5.5–14.7) per 100 child years among anemic children, while it was 2.86 (95% CI, 2.1–3.8) per 100 child years among non-anemic children (Fig 3).

### Model adequacy

**Fig 4:**
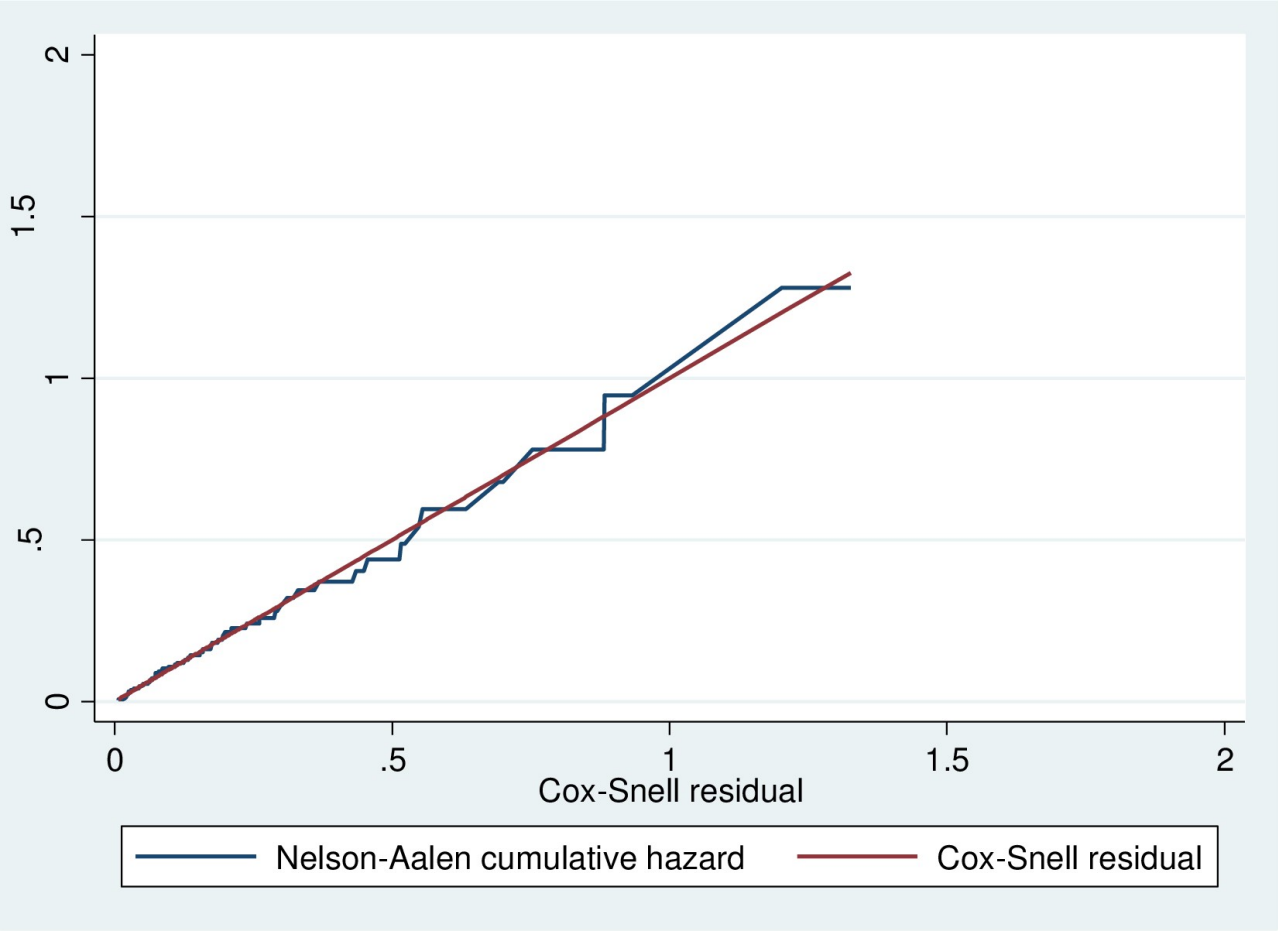
Cox-Snell residual plot showing goodness fit of Gompertz -regression model among children on ART in Wolaita Zone, southern Ethiopia, January 2010 to December 2022.

The Schoenfeld global test confirmed the proportional hazard assumption (P-value = 0.8168). Various models were compared to identify the best model, and the parametric survival test with Gompertz distribution was used to generate estimates with the lowest Akaike information criteria (AIC) (S1 File). The baseline hazard of tuberculosis has a Gompertz distribution with a shape parameter of less than one (P = -0.27, 95% CI: -0.41, -0.12), indicating a constant decrease. The shared frailty was estimated, and the theta was significantly different from zero (theta: 1.90^e-06^), but this shows that the distribution of unmeasured variables is indifferent between the hospitals and health centers. The hazard function follows the 45-degree line very closely, and it is feasible to conclude that the final model fits the data very well (**Fig 4**).

### Predictors of TB incidence in HIV-positive children on ART

In bi-variable parametric survival Gompertz regression: older children (> 10 years old), family size ≥ 5, WHO clinical stages III or IV, receiving an IPT, receiving CPT, hemoglobin level <10mg/dl, stunting, underweight, and fair or poor ART adherence were found to be a significant predictor of TB among children in ART. In the final multivariable model, age 10 to 15 years, WHO clinical stages III or IV, hemoglobin level <10mg/dl, being underweight, and fair or poor adherence level have significantly increased the incidence of TB while receiving CPT significantly decreased the incidence of TB.

Children who were classified as in WHO clinical stages III or IV had 2.3 times (AHR = 2.31, 95% CI [1.26, 4.22]) higher risk of getting TB infection than those in WHO clinical stages 1 or 2. Children whose adherence level was fair or poor for ART had 4.4 (AHR = 4.4, 95% CI [2.18, 9.05]) times more risk of developing TB compared to children with good ART adherence. Children who received CPT had a 77% (AHR = 0.23; 95% CI [0.08, 0.60]) lower risk of developing TB compared to children who did not receive CPT. Anemic (AHR = 2.87, 95% CI [1.51, 5.45]), and underweight (AHR = 2.55, 95% CI [1.45, 4.51]) children had an increased risk of developing TB compared to non-anemic and normal-weight children. Furthermore, older children (> 10 years old) were 3.5 years (AHR = 3.62; 95% CI [1.29, 10.0]) times more likely to develop TB compared to children aged <5 years (Table 3).

**Table 3:** Bivariable and multivariable parametric Gompertz regression analysis for predictors of TB among children receiving ART in Wolaita Zone between January 2010 to December 2022.

Variables	Categories		Incident TB	CHR 95%CI	AHR 95%CI
		Yes (n, %)	No (n, %)		
Age	<5	8(12.7)	55(87.3)	1	1
	6–10	40(15.4)	219(84.6)	1.18(0.55, 2.53)	1.91(0.82, 4.47)
	10–15	11(22.4)	38(77.6)	2.03(0.81, 5.05) *	3.48 (1.27, 9.48) *
Sex	Male	33(15.3)	183(84.7)	1	-
	Female	26(16.8)	129(83.2)	1.04(0.62, 1.74)	-
Residence	Urban	47(16)	246(84.0.0)	1	-
	Rural	12(15.4)	66(84.6)	0.79(0.41, 1.49)	-
Family Size	≤2	3(8.3)	33(91.7)	1	1
	3–5	24(13.6)	152(86.4)	1.90(0.57, 6.33)	1.18(0.34, 4.11)
	≥5	32(20.1)	127(79.9)	2.97(0.91, 9.73) *	1.71(0.49, 5.9)
Baseline WHO stage	Stages I or II	38(13.6)	241 (86.4)	1	1
	Stages III or IV	21(22.8)	71(77.2)	1.83(1.07, 3.1) *	2.27 (1.24, 4.15) **
Baseline CD4 count	Below threshold	41(14.0)	251(86)	1	1
	Above threshold	18(22.8)	61(77.2)	0.58 (0.33,1.05) *	0.80(0.44,1.46)
Baseline Hemoglobin	< 10 mg/dl	16(33.3)	32(66.7)	2.93(1.65, 5.21)	2.77(1.46, 5.25) **
	≥ 10 mg/dl	43(13.3)	280(86.7)	1	1
Ever taking CPT	Yes	54(15.0)	305(85.0)	0.27(0.11, 0.69) **	0.23(0.08,0.64) **
	No	5(41.7)	7(58.3)	1	1
Ever taking IPT	Yes	46(14.5)	272(85.5)	0.53(0.28,0.98) *	0.77(0.33,1.49)
	No	13 (24.5)	40(75.5)	1	1
ART adherence	Good	48(14.1)	291(85.9)	1	1
	fair and poor	11(34.3)	21(65.7)	3.27(1.7, 6.3)	4.4 (2.1, 9.0) **
Baseline weight for age	Normal	40(13.5)	257(86.5)	1	1
	Underweight	19(25.7)	55(74.3)	2.17(1.26, 3.75)	2.62(1.48, 4.62) **
Baseline height for age	Stunted	13(22.8)	44(77.2)	1.70(0.92, 3.16)	1.34 (0.68, 2.67)
	Normal	46(14.6)	268(85.4)	1	1

note *significant at a p-value <0.05 and ** significant at a p-value <0.01, WHO: World Health Organization; CPT: Cotrimoxazole preventive therapy; IPT: Isoniazid preventive therapy, cluster of differentiation, CHR: Crude hazard ratio AHR: Adjusted hazard ratio.

## Discussion

In this study, the overall incidence rate of TB among HIV-positive children on ART was 3.5 (95% CI 2.7–4.5) per 100 child years. WHO clinical stages III or IV, hemoglobin level <10 g/dL, ART adherence level fair or poor, underweight, age > 10 years, and receiving cotrimoxazole preventive therapy were the independent predictors of TB.

The finding is higher than a study conducted at Debre-Markos Referral Hospital, which reported rates of 2.63 per 100 child years [7]. This might be due to the differences in sociodemographic and baseline clinical characteristics of study participants and a failure to screen for TB before initiation of ART because this study included health centers. However, it is lower than the finding of a study conducted in southwest Ethiopia, which reported an incidence of TB of 7.9 per 100 child years [8]. Also, this finding is lower than studies conducted in north Ethiopia and Adama Referral Hospital and Medical College, which reported rates of 4.2 and 6.03 per 100 child years, respectively [9,10]. Studies from sub-Saharan African countries reported that the incidence rate of TB ranges from 1.9 to 5.2 per 100 child years [21,22].

The incidence of TB was high in the first five months after initiation of ART, at 110 per 1000 child years. The rate significantly decreased to 0.62 per 1,000 child years after five years. The trend of TB incidence was in line with a study conducted in Thailand and Zimbabwe [11,22]. The possible reason for this is that about 20% of children initiate ART with a CD4 count below 200 cells/mm3. Evidence indicated that 23.1% of patients who had initiated ART with CD4+ counts < 50 cells/mm3 developed IRIS within three months of ART initiation due to immune reconstitution, doubling TB incidence by fourfold [23,24].

In agreement with the previous literature [5,9,21], the advanced WHO clinical stage was the independent predictor of incident TB among people with HIV in this study. Children in WHO clinical stages III or IV were at higher risk of getting TB infections than those in WHO clinical stages I or II. This could be explained by the fact that declining immunity among patients with advanced WHO clinical stages precipitates the progression from latent TB infection to active TB infection [3]. Children who arrive with advanced WHO clinical stages, therefore, need special monitoring.

Evidence indicates that antiretroviral therapy has significantly reduced morbidity and mortality among people living with HIV [8]. However, their poor adherence to ART increased the risk of mortality and developing TB by 5.2 and 4-fold, respectively [7,25]. In this study, the risk of TB was 4.4 times higher among children who had fair or poor adherence to ART compared to those who had good adherence to ART. This finding was supported by a study in northern Ethiopia [7]. To achieve optimal benefits from ART, high levels of patient adherence are essential [26]. ART plays a significant role in preventing viral replication and restoring immune function, while lower adherence to ART can create a suitable environment for viral replication.

The age of children was an independent predictor of childhood tuberculosis. The risk of developing TB for children aged greater than ten years was 3.62 times higher than that for those getting TB in children aged 5 years. This finding was supported by a study conducted in the Bale zone, where older children were 1.94 times more likely to develop TB infection than children younger than 5 years [27]. This could be explained by the fact that older children were engaged in outdoor activities, which increased the risk of contact with infectious cases in the community.

Cotrimoxazole preventive therapy has reduced the incidence of TB among HIV-positive children on ART. In this study, children who received CPT had a 77% reduced risk of developing TB compared to those who did not receive CPT. A study conducted in northern Ethiopia reported that the risk of developing TB was 4.3 times higher among those who did not use CPT as compared with CPT users [9]. This could be because the effect of CPT averts the occurrence of pneumocystis pneumonia(PCP) [28]. PCP is a common HIV-associated opportunistic infection that worsens immunosuppression and increases the likelihood of TB infection [29].

Anemia impairs the host’s defense against TB infection and increases susceptibility to infection [30,31]. The risk of developing TB for anemic children was 2.87 times higher compared to non-anemic children. This finding was in line with studies conducted in a different part of Ethiopia [9,10,16]. This may be due to azidothymidine (AZT) being known to be one of the most common causes of megaloblastic anemia, which is, in turn, closely associated with pancytopenia [32]. This condition imposes a double burden on children with HIV and makes them vulnerable to infections, including TB.

Furthermore, undernutrition and HIV are closely interlinked, creating a vicious cycle [33]. Undernutrition can be a risk factor for developing severe diseases with worse prognoses and impaired immune recovery in HIV-infected children [34,35]. Undernutrition was associated with decreased CD4% resistant recovery rates after 48 weeks of ART [36]. In this study, underweight children had a 2.55-fold increased risk of developing TB compared to normal-weight children. This finding was consistent with studies conducted in northern and central Ethiopia [9,10]. The linkage between malnutrition and tuberculosis has long been recognized. Malnutrition may predispose people to develop clinical diseases, and tuberculosis can contribute to malnutrition [34].

The current study found that IPT had not significantly decreased the incidence of tuberculosis among HIV-positive children, which is similar to previous studies conducted in Ethiopia [6,7,16]. This may be because child-initiated IPT with active TB in resource-limited settings lacks diagnostic modalities, challenges HIV-positive children’s diagnosis, and may impact the effectiveness of IPT in reducing TB through poor adherence, inadequate counseling, and withdrawal due to side effects without consulting healthcare providers [37]. The possible limitation of this study is that, since it is a chart review, it failed to consider a broad range of factors like housing characteristics and environmental and family history of smoking-related factors. Also, children who started ART at the beginning and who developed TB in less than one month were excluded from the study. This may be an underestimation of the incidence of tuberculosis.

## Conclusion

The incidence of tuberculosis among children on ART was high. WHO clinical stages III or IV, hemoglobin level <10 g/dL, ART adherence fair or poor, underweight, age >10 years, and receiving cotrimoxazole preventive therapy were the independent predictors of TB occurrence. Therefore, counseling on ART adherence, early diagnosis, and prompt treatment of anemia and malnutrition are recommended to decrease the risk of TB infection.

## Supporting information

S1 File Comparison of the models. (DOCX)

S1 Data (DTA)

## Abbreviations

AHR: Adjusted hazard ratio
AIC: Akaike’s information criterion
AIDS: Acquired Immunodeficiency Syndrome
ART: Antiretroviral therapy
BIC: Bayesian information criterion
CD4: Cluster of Differentiation 4
CPT: Cotrimoxazole prophylactic therapy
IPT: Isoniazid preventive therapy
