# Supplementary material for: Incidence and predictors of tuberculosis among children receiving antiretroviral therapy in the Wolaita Zone: A retrospective cohort study
Source: PLoS One. 2023 Sep 21;18(9):e0291502. doi: 10.1371/journal.pone.0291502 (PMC10513190; doi:10.1371/journal.pone.0291502)
Supplement: S1 File — (DOCX) [file pone.0291502.s001.docx]

Comparison of survival analysis models

| Model | Log-likelihood | AIC | BIC |
| --- | --- | --- | --- |
| Cox | -304.13 | 632.26 | 679.25 |
| Gompertz | **-211.17** | **450.34** | **505.16** |
| Exponential | -219.09 | 464.19 | 515.1 |
| Weibull | -214.88 | 457.7 | 512.5 |
| Loglogistic | -214.24 | 456.48 | 511.3 |
| Lognormal | -213.5 | 455.1 | 510.0 |
